# Comprehensive treatment experience of anal squamous cell carcinoma from a tertiary cancer center in South China

**DOI:** 10.1002/cam4.4433

**Published:** 2021-11-24

**Authors:** Yan Yuan, Wei‐Hao Xie, Rong‐Zhen Li, Hui Chang, Zhi‐Fan Zeng, Yuan‐Hong Gao, Qiao‐Xuan Wang, Wei‐Wei Xiao

**Affiliations:** ^1^ State Key Laboratory of Oncology in South China Collaborative Innovation Center for Cancer Medicine Guangzhou PR China; ^2^ Department of Radiation Oncology Sun Yat‐sen University Cancer Center Guangzhou PR China

**Keywords:** anal squamous cell carcinoma, chemoradiotherapy, induction chemotherapy, prognosis

## Abstract

**Background:**

Anal squamous cell carcinoma (ASCC) is a rare malignant tumor with increasing incidence. The goal of our study was to analyze the treatment outcome and prognostic factors of ASCC in South China in the past half‐century.

**Methods:**

This study retrospectively included 59 patients with ASCC admitted from 1975 to 2018 in Sun Yat‐sen University cancer center. The clinical records and follow‐up information of all patients were collected. Survival analysis and univariate and multivariate regression analyses were performed using the “survival” and “survminer” packages of R software.

**Results:**

In 59 patients, 5 patients had distant metastasis at diagnosis. Among 54 M0 stage patients, 33 patients received chemoradiotherapy (CRT), 19 patients received local surgery, and 2 patients refused curative treatment and received the best supportive treatment (BST). The most common grade 3–4 acute toxicities during treatment were myelosuppression and radiation dermatitis. The median follow‐up time was 32 months. For the whole group, the 3‐year and 5‐year overall survival (OS) rates and disease‐free survival (DFS) were 71.1% and 63.6%, and 73.4% and 69.0%, respectively. Multivariate regression analysis showed that the T3–4 stage was an independent prognostic risk factor for OS, progression‐free survival (PFS), and DFS. And M1 was an independent prognostic risk factor for PFS and DFS. Patients in stage M0 mainly treated with CRT had better local control than those mainly treated with surgery (*p* = 0.027). For M0 patients, induction chemotherapy combined with CRT tends to prolong OS compared with CRT alone (*p* = 0.26). The 3‐year colostomy‐free survival for the whole group was 81.1%.

**Conclusions:**

CRT is recommended as the first choice for the treatment of M0 stage ASCC. Induction chemotherapy may bring better survival benefits for some patients. Patients with ASCC in China seem to have a better local control rate, which suggested different treatment strategies may be needed in China.

## Graphical abstract

CRT is recommended as the first choice for the treatment of M0 stage ASCC. Induction chemotherapy may bring better survival benefits for some patients. The different failure modes of disease control required different treatment strategies in China.

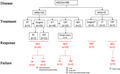

## 1 INTRODUCTION

Anal squamous cell carcinoma (ASCC) is a rare malignant tumor with an increasing incidence, accounting for about 1%–2% of all digestive tract malignant tumors.^1^, ^2^ High‐risk human papillomavirus (HPV) infection is strongly associated with ASCC, and the infection rate of HPV in ASCC was about 72%–90% in the United States and Europe.^3^, ^4^, ^5^ Previous studies indicated that the HPV‐52, HPV‐16, and HPV‐58 accounted for the top three common genotypes in the high‐risk HPV infection among women in China.^6^ HPV infection was not only associated with the risk of ASCC but also played a significant role in their sensitivity to chemotherapy and radiotherapy.^7^ A history of receptive anal intercourse, a history of gynecological tumors, and human immunodeficiency virus infection were also included in the risk factors of ASCC.

Before the 1970s, the main treatment of anal squamous cell carcinoma was extensive abdominoperineal resection (APR).^8^ With the gradual understanding of the biological behavior of ASCC, the treatment mode has changed fundamentally. Currently, chemoradiotherapy (CRT) as the main treatment of ASCC, could achieve radical treatment and improve the survival rate, with the retention of anus to improve patients’ quality of life.^9^, ^10^ Local resection (LR) is mainly recommended for T1N0M0 perianal cancer and salvageable surgery is needed upon local tumor recurrence after CRT for ASCC.^9^ Local recurrence and distant metastasis occurred in 20%–30% and 10% locally advanced ASCC after CRT in the United Kingdom, Australia, Norway, and the United States.^11^, ^12^ In addition, about 10% of the patients were diagnosed with advanced metastatic ASCC, and the 5‐year overall survival (OS) rate was only about 30% in the United States.^13^, ^14^

The incidence of ASCC was also very low in China and the treatment modalities always followed international guidelines. However, few works of literature focus on the treatment results of ASCC in China. Lu et al described the epidemiological characteristics of ASCC and prognostic factors of survival outcomes of 144 ASCC patients diagnosed between 2007 and 2018 from 11 cancer hospitals in southern China.^15^ Results suggested that age >50 years, advanced AJCC stage, and lymph node positivity were correlated with poor survival of ASCC patients. The estimated 5‐year OS and relapse‐free survival (RFS) were 82.8% and 79.4%, respectively. This study only observed the epidemiology and treatments effect of ASCC in China after 2007. The majority of patients were treated with CRT, and the specific failure pattern of treatment was not specifically analyzed. In the present study, we particularly described the results of the administration of ASCC patients over the past half‐century at a comprehensive tertiary cancer center in South China.

## 2 METHODS

### 2.1 Patient population and staging system

This study screened patients of ASCC treated in Sun Yat‐sen University Cancer Center from 1 January 1975 to 31 December 2018. The study included all patients with clinical, pathological, and imaging diagnoses of ASCC. Medical records, image data, and pathological reports of all ASCC patients were collected. HPV testing was performed in five patients and two were positive. Previous medical history was noted that two patients had cervical cancer and one patient had vulvar cancer cured before diagnosis of ASCC. The initial clinical stage of all patients was re‐accessed according to the eighth edition of the Union for American Joint Cancer Committee (AJCC) TNM staging system. According to the eighth edition of the AJCC Cancer Staging Manual, we divided patients into two categories as the anal canal squamous cell carcinoma and the perianal squamous cell carcinoma. This study was approved by the Ethics Committee of our cancer center (IRB B2020‐125‐01).

### 2.2 Clinical characteristics collection

The gender, age, smoking history, and gynecological tumor history of all patients were collected. The information of tumor location, tumor grade, clinical TNM stage, laboratory examination, treatment regimens, radiation therapy technology, surgical approach, and treatment‐related adverse events was also systemically recorded. The assessment of acute adverse events which occurred during or 1 month after CRT was re‐performed according to the Common Terminology Criteria for Adverse Events (CTCAE) (version 4.03). The evaluation of postoperative complications was based on the Clavien–Dindo classification.^16^

### 2.3 Survival definition and follow‐up

The censoring date for the survival analysis was 31 April 2020. Overall survival (OS) was defined as the time from the diagnosis date to death for any reason. Progression‐free survival (PFS) definition was defined as the time from the diagnosis date to tumor progression or death. Disease‐free survival (DFS) was defined as the time from the diagnosis date to tumor recurrence which was primarily used to evaluate patients with no evidence of disease (NED) status after treatments. Relapse‐free survival (RFS) was defined as the time from the diagnosis date to the local recurrence of the tumor. Metastasis‐free survival (MFS) was defined as the time from the diagnosis date to the occurrence of distant metastasis. Colostomy‐free survival (CFS) was defined as the time from the diagnosis date to the colostomy date. Survival follow‐up was conducted by outpatient service or household registration system. The last follow‐up time point was 30 April 2020.

### 2.4 Statistical analysis

Descriptive statistics were used to report the data obtained from the medical records. Medians with interquartile ranges (IQRs) were used to record the continuous data. Numbers with percentages (%) were used to record the categorical data. The study has included patients treated over a long period (1975–2018). During this period, diagnostic, therapeutic, and supportive care practices have changed significantly. Before December 2008, the initial treatment for ASCC was surgery which was changed to CRT after 2008 in our center. Therefore, we divided data between those treated prior to 2008 and 2009–2018 and compare these two groups. Survival analysis and univariate and multivariate regression analyses were performed using the “survival” (2.44‐1.1) and “survminer” (0.4.6) package in R software (3.6.1), and the survival analysis curve was plotted. The direct correlation of covariates was calculated using Hmisc R package (4.2‐0). When the correlation coefficient is greater than 0.8, it is considered that there is a serious correlation between covariates. Based on the results of univariate analysis (*p* < 0.2) and correlation analysis (*R* < 0.8), those variables were included in the multivariate analysis.

## 3 RESULTS

### 3.1 Clinical characteristics and treatments

**FIGURE 1:**
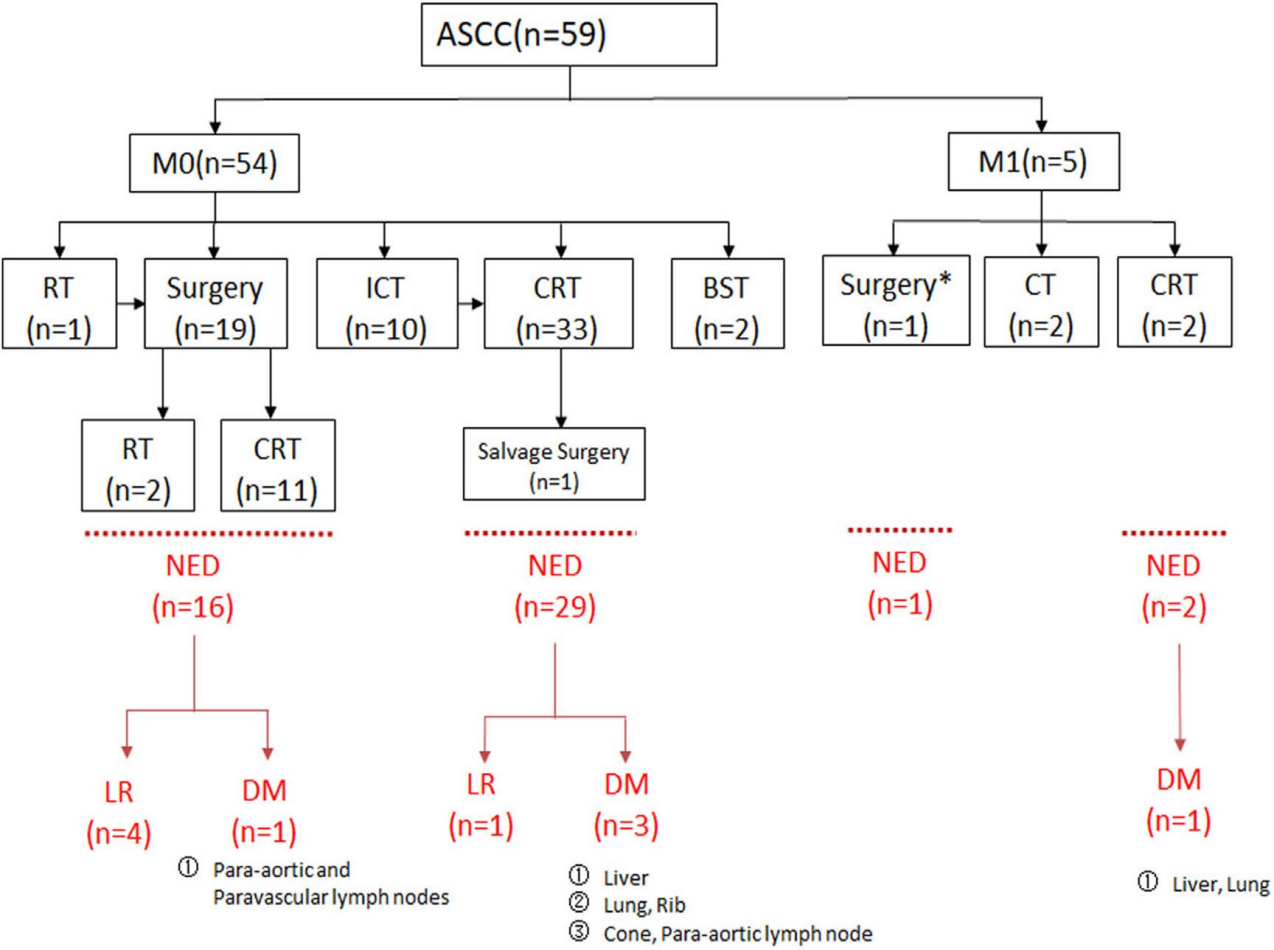
The treatments and efficacy of all patients. Abbreviations: ICT, induction chemotherapy; CT, chemotherapy; CRT, chemoradiotherapy; RT, radiotherapy; NED, no evidence of disease; LR, local recurrence; DM, distant metastasis; Note: *, abdominoperineal resection with intrahepatic anhydrous alcohol injection

The study profile is shown in Figure 1. A total of 59 patients with ASCC were included in the study. The clinical characteristics of all patients are listed in Table 1. The median age was 54 years (IQR: 46–62) old with female patients (48, 81.4%) constituting the majority of the cohort. Anal canal site (51, 86.4%) was more common than perianal site (8, 13.6%). The most common clinical T stage was T2 (24, 40.7%) with diameters of primary tumors of more than 2 cm but not more than 5 cm. Regional lymph node metastasis (41, 69.5%) was detected in the majority of patients. Only five (8.5%) patients have distant metastasis at preliminary diagnosis. No patient had HIV infection. Only two patients were documented HPV positive with most patients did not undergo HPV testing. Three (5.1%) patients had a history of gynecological oncology as mentioned before.

**TABLE 1:** Baseline pathoclinical characteristics of the 59 patients with anal cancer

Characteristic	*N* (%)
Median age at diagnosis (range)	54 (17–88)
Gender	
Male	11 (18.6%)
Female	48 (81.4%)
Location of tumor	
Anal canal	51 (86.4%)
Perianal	8 (13.6%)
Tumor differentiation	
High‐grade intraepithelial neoplasia	7 (11.9%)
High	7 (11.9%)
Moderate	19 (32.1%)
Low	16 (27.1%)
Unknown	10 (17.0%)
cT stage	
T1	6 (10.2%)
T2	24 (40.7%)
T3	12 (20.3%)
T4	15 (25.4%)
Tx	2 (3.4%)
cN stage	
N0	17 (28.8%)
N1	41 (69.5%)
Nx	1 (1.7%)
cM stage	
M0	54 (91.5%)
M1	5 (8.5%)
Clinical stage (AJCC eighth ed.)	
I	4 (6.8%)
II	10 (15.3%)
III	38 (64.4%)
IV	5 (8.5%)
Unknown	2 (20.3%)
Immunosuppression	
HIV negative	59 (100.0%)
HPV tumor status	
Positive	2 (3.4%)
Negative	3 (5.1%)
Unknown	54 (91.6%)
Smoking history	
Yes	10 (16.9%)
No	49 (83.1%)
Gynecological oncology history	
Cervical cancer	2 (3.4%)
Vulvar cancer	1 (1.7%)
No	56 (94.9%)

Abbreviations: cM stage, clinical M stage; cN stage, clinical N stage; cT stage, clinical T stage; HBV, hepatitis B virus; HIV, human immunodeficiency virus; HPV, human papillomavirus.

Since the patients enrolled in this study for nearly half a century, the treatment mode (Table 2) also gradually changed with time migration with new evidence of diagnosis and treatment. As shown in Figure S1, 19 patients underwent surgery‐based treatment including 8 patients underwent APR surgery, 8 patients underwent LR, and 3 patients underwent palliative surgery. And 33 patients underwent CRT‐based treatment in M0 stage patients with ASCC. Ten patients received induction chemotherapy (ICT) before concurrent chemoradiotherapy (CCRT), and the main induction chemotherapy regimen was docetaxel and cisplatin (TP) (9/10, 90%). There were various kinds of chemotherapy regimens in CCRT, the most commonly used was cisplatin and fluorouracil (PF) regimen (16/54, 29.6%). The technology and dose of radiotherapy are shown in Table 2. The dose of gross tumor volume (GTV) was 45–66 Gy/25–33 fractions. The dose of clinical target volume (CTV) was 41.4–45 Gy/23–25 fractions. One patient underwent salvage APR surgery for local recurrence at 7 months after CCRT. Two M0 patients with ASCC diagnosed before 2003 refused surgery due to economic conditions and received the best supportive treatment (BST). One patient with hepatic metastasis of ASCC underwent APR combined with intrahepatic anhydrous alcohol injection in 1998. Palliative chemotherapy and CRT were adopted for the remaining four M1 patients.

**TABLE 2:** The treatment strategy of M0 ASCC patients (*n* = 54)

	*N* (%)
Neoadjuvant therapy	
ICT + CCRT	10 (18.5%)
CCRT	33 (61.1%)
Regimes of ICT	
DPF	1
TP	9
Regimes of CRT	
Capecitabine	3 (5.6%)
S−1	1 (1.9%)
5‐Fu	3 (5.6%)
DDP	8 (14.8%)
PF	16 (29.6%)
TP	6 (11.1%)
Capeox	3 (5.6%)
FOLFOX	1 (1.9%)
Unknown	2 (3.7%)
Radiation therapy technology	
X‐ray + Co60	1 (1.9%)
3D‐CRT	4 (7.4%)
IMRT	37 (68.5%)
IGRT + three‐dimensional brachytherapy	1 (1.9%)
Unknown	2 (3.7%)
Radiation therapy plan	
GTV dose	45–70 Gy
GTV fraction	25–35
CTV dose	41.4–51 Gy
CTV fraction	23–30
Surgery patterns	
APR	9 (16.7%)
LR	8 (14.8%)
Palliative surgery	3 (5.6%)

Abbreviations: 3D‐CRT, three‐dimensional conformal radiation therapy; 5‐Fu, fluorouracil; APR, abdominoperineal resection; CCRT, concurrent chemoradiotherapy; CTV, clinical target volume; DDP, cisplatin; DPF, cisplatin, docetaxel, and fluorouracil; FOLFOX, oxaliplatin, fluorouracil, and calcium leucovorin; GTV, gross tumor volume; ICT, induction chemotherapy; IGRT, image‐guided radiation therapy; IMRT, intensity‐modulated radiotherapy; LR, local resection; TP, docetaxel and cisplatin.

### 3.2 Short‐term efficacy and adverse events

In the entire study, a total of 49 patients retained anus with guaranteed quality of life. Forty‐five M0 ASCC patients achieved clinical NED status after surgery or CRT in the short‐term efficacy evaluation. Three M1 patients achieved NED status after system treatments. The 3‐year CFS rate was 81.1% for the whole group. The information about the treatment‐related toxicities is shown in Table 3. No postoperative complications occurred in the study. The main grade 3–4 acute toxicity related to treatment in the whole group was myelosuppression, with mild intestinal toxicity, skin toxicity, and peripheral nerve toxicity. No patients died during the treatment.

**TABLE 3:** Toxicities of treatments in the 59 patients with anal cancer

Myelosuppression	*N* (%)
Grade 0–2	48 (81.4%)
Grade 3–4	11 (18.6%)
GI toxicities	
Grade 0–2	58 (98.3%)
Grade 3–4	1 (1.7%)
Peripheral neurotoxicity	
Grade 0–2	58 (98.3%)
Grade 3–4	1 (1.7%)
Radiation enteritis/dermatitis	
Grade 0–2	53 (89.8%)
Grade 3–4	6 (10.2%)

Abbreviation: GI, gastrointestinal.

### 3.3 Longtime survival

**FIGURE 2:**
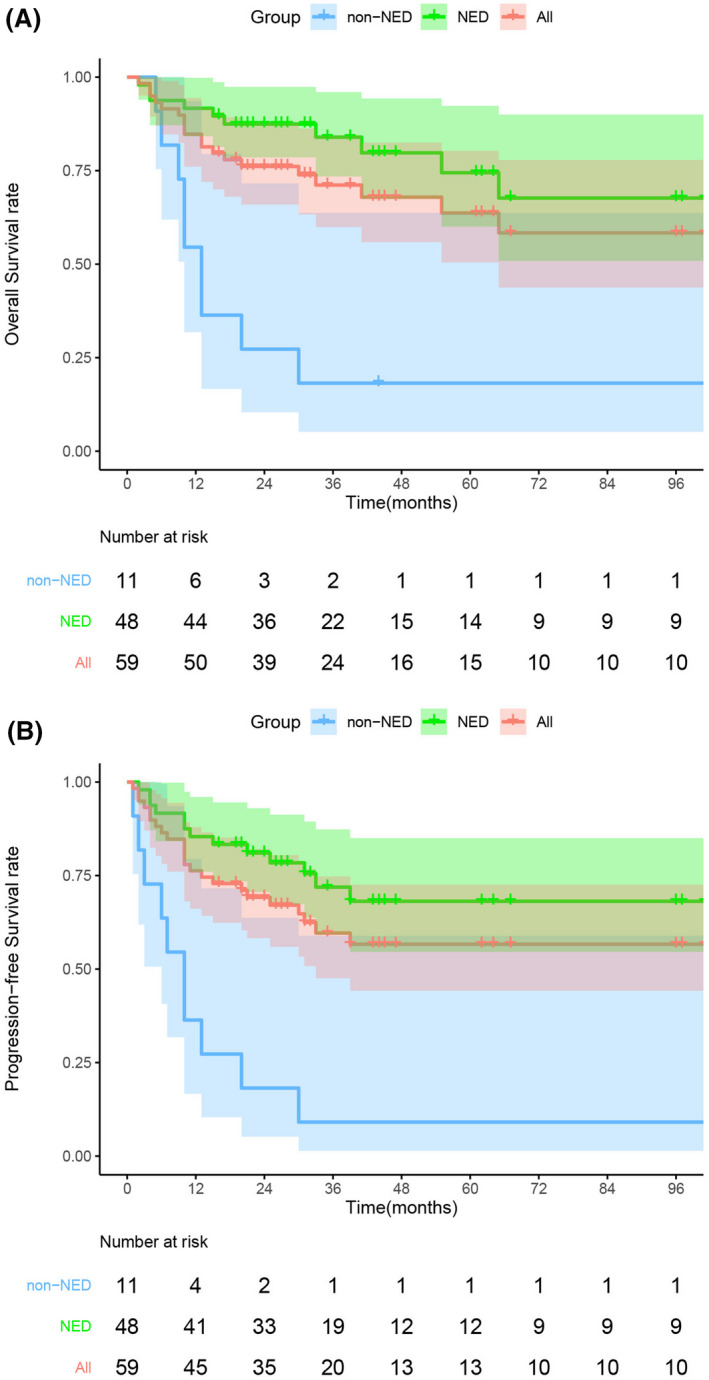
Survival curves. Overall survival curves (A) and Progression‐free survival curves (B) of the patients with anal cancer. Abbreviations: NED, no evidence of disease

The median follow‐up was 32 months (ranging from 2 to 265 months, IQR: 19–62 months). The survival rates of different groups are shown in Table 4. In the present study, the 3‐year and 5‐year OS rates were 71.1% and 63.6%, and the 3‐year and 5‐year PFS rates were 60.70% and 57.3%, respectively (Figure 2).

**TABLE 4:** Survival rate

Survival rate	All (%)	NED (%)	Non‐NED (%)
CFS			
3‐year	81.10	79.10	90.09
5‐year	81.10	79.10	90.09
OS			
3‐year	71.10	84.00	18.20
5‐year	63.60	74.40	18.20
PFS			
3‐year	60.70	73.40	9.10
5‐year	57.30	69.00	9.10
RFS			
3‐year	90.80	90.00	–
5‐year	86.30	85.00	–
MFS			
3‐year	84.10	87.10	55.60
5‐year	84.10	87.10	55.60

Abbreviations: CFS, colostomy‐free survival reported for number of participants who did not develop local recurrence or require resection with colostomy; MFS, metastasis‐free survival; NED, no evidence of disease; OS, overall survival; PFS, progression‐free survival; RFS, relapse‐free survival.

Patients with NED had significant higher OS and PFS as shown in Table 4 and Figure 2. The 5‐year OS and PFS rates for patients achieved NED were 74.4% and 69.0%, while the 5‐year OS and PFS rates for patients did not achieve NED were only 18.2% (*p* < 0.001) and 9.1% (*p* < 0.001).

The 3‐year and 5‐year OS rates for M0 group were 74.2% and 66.4%, and the 3‐year and 5‐year DFS rates were 73.4% and 69.0%, respectively. However, the 3‐year and 5‐year survival rates of M1 patients with ASCC were only 30% (Table 5).

**TABLE 5:** Univariate and multivariable Cox analyses of prognostic factors for OS, PFS, and DFS in 59 patients with anal cancer (OS, *p* = 0.0002, χ^2^ = 24.09; PFS, *p* = 0.0012,χ^2^ = 20.17; DFS, *p* = 0.0053,χ^2^ = 14.73) (OS,PFS, *n* = 57; DFS, *n* = 46)

Overall survival variable	5‐year survival rate	Univariate analysis		Multivariate analysis		HR (95%CI)
		*N*	*p*	*N*	*p*	
Gender (female vs. male)	66.2% vs. 53.0%	48 vs. 11	0.491	–	–	–
Age (≤65 years vs. >65 years )	68.0% vs. 53.7%	46 vs. 13	0.292	–	–	–
Smoking history (no vs. yes)	67.3% vs 48.0%	49 vs 10	0.718	–	–	–
Site (anal canal vs. perianal)	68.8% vs. 33.3%	51 vs. 8	**0.016**	**49 vs. 8**	0.064	3.389(0.929–12.360)
Diagnose years (1975–2008 vs. 2009–2018)	42.9% vs. 69.3%	14 vs. 45	**0.025**	**13 vs. 44**	0.134	0.421(0.135–1.307)
Differentiation (poor vs. good)^a^	67.2% vs. 67.5%	35 vs. 14	0.719	–	–	–
Tumor diameter (T1–2 vs. T3–4)	83.7% vs. 44.2%	30 vs. 27	**0.002**	**30 vs. 27**	**0.019**	4.156 (1.269–13.612)
cN stage (N0 vs. N1)	71.9% vs. 58.2%	17 vs. 41	0.070	–	–	–
cM stage (M0 vs. M1)	66.4% vs. 30.0%	54 vs. 5	**0.021**	**52 vs. 5**	0.119	3.269(0.737–14.507)
cTNM stage (I–II vs. III–IV)	90.9% vs. 57.5%	14 vs. 43	**0.019**	**14 vs. 43**	0.194	4.000(0.492–33.172)
Initial treatment (surgery vs. CRT)	62.2% vs. 66.8%	20 vs. 35	0.279	–	–	–

Progression‐free survival variable	5‐year survival rate	Univariate analysis		Multivariate analysis		HR (95%CI)
		*N*	*p*	*N*	*p*	
Gender (female vs. male)	59.5% vs. 45.5%	48 vs. 11	0.360	–	–	–
Age (≤65 years vs. >65 years)	56.3% vs. 59.3%	46 vs. 13	0.953	–	–	–
Smoking history (no vs. yes)	60.5% vs. 40.0%	49 vs. 10	0.201	–	–	–
Site (anal canal vs. perianal)	61.2% vs. 33.3%	51 vs. 8	0.146	49 vs. 8	0.079	2.741(0.891–8.464)
Diagnose years (1975–2008 vs. 2009–2018)	35.7% vs. 64.7%	14 vs. 45	0.063	13 vs. 44	0.389	0.645(0.238–1.750)
Differentiation (poor vs. good)^a^	60.4% vs. 62.5%	35 vs. 14	0.784	–	–	–
Tumor diameter (T1–2 vs. T3–4)	76.6% vs. 43.5%	30 vs. 27	**0.003**	**30 vs. 27**	**0.019**	**3.329(1.215–9.120)**
cN stage (N0 vs. N1)	72.2% vs. 54.4%	17 vs. 41	0.066	–	–	
cM stage (M0 vs. M1)	61.2% vs. 0.0%	54 vs. 5	**0.003**	**52 vs. 5**	**0.032**	**4.099(1.129–14.884)**
cTNM stage (I–II vs. III–IV)	83.6% vs. 54.1%	14 vs. 43	**0.025**	**14 vs. 43**	0.196	2.767(0.591–12.954)
Initial treatment (surgery vs. CRT)	45.7% vs. 70.4%	20 vs. 35	0.188	–	–	–

Disease‐free survival variable	5‐year survival rate	Univariate analysis		Multivariate analysis		HR (95%CI)
		*N*	*p*	*N*	*p*	
Gender (female vs. male)	69.8% vs. 62.5%	40 vs. 8	0.493	–	–	–
Age (≤65 years vs. >65 years)	68.4% vs. 70.0%	38 vs. 10	0.887	–	–	–
Smoking history (no vs. yes)	72.5% vs. 50.0%	40 vs. 8	0.098	38 vs. 8	0.250	2.677(0.500–14.322)
Site (anal canal vs. perianal)	70.2% vs. 75.0%	43 vs. 4	0.918	–	–	–
Diagnose years (1975–2008 vs. 2009–2018)	44.4% vs. 74.7%	9 vs. 39	**0.046**	38 vs. 8	0.238	0.377(0.074–1.909)
Differentiation (poor vs. good)^a^	69.5% vs. 81.8%	29 vs. 11	0.507	–	–	–
Tumor diameter (T1–2 vs. T3–4)	82.5% vs. 62.2%	27 vs. 19	0.055	27 vs. 19	**0.022**	**5.345(1.276–22.389)**
cN stage (N0 vs. N1)	76.7% vs. 70.3%	16 vs. 31	0.297	–	–	–
cM stage (M0 vs. M1)	71.8% vs. 33.3%	45 vs. 3	**0.008**	43 vs. 3	**0.003**	**22.388(2.961–169.260)**
cTNM stage (I–II vs. III–IV)	83.6% vs. 71.2%	14 vs. 32	0.216	–	–	–
Initial treatment (surgery vs. CRT)	57.1% vs. 79.5%	16 vs. 31	0.171	–	–	–

Statistically significant values are bolded (*p* < 0.05).

^a^ Differentiation, good, high‐grade intraepithelial neoplasia and high grade; bad, low grade and moderate grade.

One case died of cancer, but the failure pattern of specific diseases was not clear due to the passage of time. The recurrence and metastasis sites are shown in Figure 1. The 5‐year RFS and MFS were 86.30% and 84.1% for the whole group. In the M0 group, 45 patients achieved NED status, and among them 5 patients had local recurrence and 4 patients had distant metastasis afterward. Patients in stage M0 mainly treated with CRT had better local control than those mainly treated with surgery (5‐year RFS: 96.7% vs. 66.9%, *p* = 0.027). In the M1 group, NED was also achieved in three patients after treatment.

In the univariate analysis (Table 5), anal canal, diagnose years between 2009 and 2018, tumor diameter <5 cm, and M0 stage were significantly associated with favorable OS in the whole group. Tumor <5 cm, M0 stage, and early cTNM stage (I–II) were significantly associated with favorable PFS and diagnosis years (2009–2018) and M0 stage were significantly correlated with favorable DFS. The results of the correlation matrix (Table S1) identified strong correlations (*R* = 0.91) between two variables (cN stage group and cTNM stage group). Then we subjected the cTNM stage group to the multivariate analysis, but not cN stage. In the multivariate analysis, the tumor size remained the independent prognostic factor for OS, PFS, and DFS for ASCC. Distant metastasis at diagnosis was an independent prognostic factor for PFS and DFS.

## 4 DISCUSSION

Worldwide, ASCC was a rare malignant tumor with increasing incidence. A similar trend could be detected in our study with significantly more ASCC patients were diagnosed after 2011 (Figure S2). The main risk factors for anal cancer include HPV infection, history of anal intercourse, and history of sexually transmitted diseases, cervical cancer, perineal tumor, immunosuppression, and smoking. In Europe, the incidence of HPV infection in anal canal carcinoma was 90.7% and most of them were HPV‐16.^17^ Our statistical results showed that the positive rate of HPV16/18 in anal canal carcinoma was 90%, and there was a retrograde upward infection of proximal colorectum.^18^ Unfortunately, the majority of patients in our study were not tested for HPV. The higher incidence in females than males in our study is consistent with previous studies.^15^, ^19^ The different incidence of ASCC in genders might be related to the inconsistency of HPV infection rate between men and women. A previous study reported that sex steroids could affect mucosal immunity, with particular reference to HPV infection.^20^ Meanwhile, the majority of patients with ASCC were diagnosed as locally advanced stage though the occurrence site of the anal tumor was easily palpated by physical examination. All these suggest that early screening of ASCC is necessary, especially for women with HPV positivity in our country.

Previous research by Nigro ND et al reported preoperative CRT in three patients, the results showed that two patients achieved complete pathological remission and one patient achieved the long‐term DFS.^8^ This study suggested CRT might be curative for anal cancer without surgery. In the current study, patients who were diagnosed after 2008 mainly received curative CRT and PF is the most common used chemotherapy in our cancer instead of mitomycin (MMC) and 5‐fluorouracil (5‐Fu) (MF). Prospective randomized controlled studies carried by the United Kingdom Coordinating Committee for Cancer Research (UKCCCR) and European Organization for Research and Treatment of Cancer (EORTC) laid the first‐line treatment status of CRT with results of longer survival and lower recurrence rates than radiotherapy alone.^10^, ^11^ MF was used as chemotherapy regimens in both studies. Meanwhile, the NCCN Guidelines for ASCC recommended that MF, mitomycin/ capecitabine, PF combined with radiotherapy could be the primary treatment for a locoregional disease.^9^ However, significant nephrotoxicity, pulmonary toxicity, and bone marrow suppression limited the clinical use of mitomycin.^21^, ^22^, ^23^ A phase III randomized intergroup study revealed the toxicity was greater in the 5‐Fu plus MMC group than in the 5‐Fu group (grade IV, 23% vs. 7%; grade V, 2.7% vs. 0.7%) for patients with anal cancer.^24^ Similar results were reported in the RTOG 98–11 study with higher hematologic grade 3 or 4 toxicity in the FU/MMC arm compared with the FU/DDP arm (61.8% vs. 42%).^25^ Therefore, the mainstream chemotherapy regimens were PF and docetaxel plus cisplatin (TP) in China.^15^, ^26^, ^27^ A long‐term result revealed that the actuarial 10‐year OS and DFS rates for the PF group were equivalent to the 5‐fluorouracil plus MMC (MF) group (OS 54% vs. 52%, *p* = 0.32, DFS 49% vs. 53%, *p* = 0.92).^28^ Meanwhile, the 5‐year cumulative colostomy rate was not significantly different between the PF group and the MF group (22% vs. 29%; *p* = 0.28). Based on the results of the phase III UK ACT II trial, the replacement of mitomycin with cisplatin in chemoradiotherapy did not affect the rate of complete response of ASCC.^29^ These results were inconsistent with the results of the US Gastrointestinal Intergroup trial RTOG 98–11.^25^ Further researches were needed to explore the curative effect of the PF regimen compared with the MF regimen.

According to the previous literature, local ASCC patients could achieve a local control rate of 70%–90%, a 5‐year DFS rate of 60%–70%, and a 5‐year OS rate of 60%–75% through curative CRT in European and American countries.^29^, ^30^ The rates of treatment failure which contained pelvic recurrence, distant metastasis with pelvic recurrence, and distant metastasis with no local recurrence were 64%, 14%, and 22%, respectively.^29^ Pelvic recurrence remains the leading cause of disease control failure. Nevertheless, few studies focus on the treatment and prognosis of ASCC in China currently. Lu Y et al reported the clinical and epidemiological characteristics of 144 ASCC patients from multicenter in Southern China after 2007 as we mentioned before.^15^ Li J et al evaluated the efficacy and safety of DDP/capecitabine (XP) with intensity‐modulated radiation therapy (IMRT) in 11 ASCC patients in Southwest China from January 2017 to June 2019.^26^ In our study, we collected all the cases admitted since the establishment of our center, and analyzed the treatment and survival of all patients in detail. The 5‐year OS and PFS rates for all patients were 63.6%, and 57.3%. The 5‐year OS and DFS rates for M0 group were 66.4% and 69.0%, respectively. The slightly lower 5‐year survival rates in our study might be correlated with the larger annual span of our cases. Due to the poor correlation between the diagnosis year before 2008 and OS and DFS, it may be due to the fact that surgery was the main treatment for ASCC in our center before 2008, and the low economic level and health awareness of patients led to poor medication compliance. As the diagnosis year before 2008 was correlated with poor OS and DFS, which might be due to the treatment of ASCC in our center was mainly surgery before 2008, and the low level of economic level and health awareness of patients results in poor medication compliance. NED is of vital importance for patients to get the long‐term survival, which is consistent with reports from ACT II study.^32^

However, the 3‐year and 5‐year RFS were higher in our study, which were 90.8% and 86.30%, than the rates reported in previous researches.^15^, ^19^ This might be associated with the high percentage of patients who received surgery at initial treatment. The 3‐year and 5‐year CFS rates were significantly higher than rates reported in the literatures for ASCC patients undergoing CCRT. The probability of distant metastasis in this study was similar to previous studies. In summary, it might suggest that the local recurrence may not be the main cause of treatment failure in China. Therefore, stronger systemic treatment may be more meaningful for disease control.

Currently, ICT was still controversial in the management of locally ASCC worldwide. Although the ACCORD03 and RTOG9811 studies did not show significant benefits of ICT for the long‐term survival of locally ASCC.^31^ In 2005, a Swedish study suggested that platinum‐based neoadjuvant chemotherapy could improve the CR rate (92% vs. 76%, *p* < 0.01) and 5‐year OS rate (63% vs. 44%, *p* < 0.05) for anal cancer with *T* ≥ 4 cm or *N* + compared with radiotherapy ± bleomycin.^32^ A retrospective study conducted by Moureau‐Zabotto L et al revealed that patients who received ICT had a statistically significant better 5‐year CFS in France.^33^ In the present study, 10 patients received ICT before CCRT and might contribute to a better survival (Figure S3). As most patients with ASCC were diagnosed as locally advanced, we believe that more active systemic treatment was necessary. Therefore, ICT might be an alternative treatment for bulk and locally advanced ASCC.

Nowadays immunotherapy has shown good antitumor immune response in various solid carcinomas, such as melanoma, lung cancer, and head and neck squamous cell carcinoma.^34^ In 2017, Ott PA et al assessed the safety and efficacy of pembrolizumab for the cohort of patients with advanced anal carcinoma.^35^ Results showed a safety and satisfied disease control rate of 58%. Subsequently, the NCI9673 study reported that nivolumab monotherapy could be a promising approach for refractory metastatic ASCC.^36^ A case report in the United States suggested that a refractory ASCC reached near complete response using modified docetaxel, cisplatin, and 5‐Fu chemotherapy after immunotherapy.^37^ All these reports suggest that immunotherapy and traditional chemotherapy may have synergistic antitumor effects for ASCC. Accordingly, our group intended to carry out a prospective, single‐arm clinical trial to assess the efficacy of ICT combined with immunotherapy sequential radiotherapy concurrent with immunotherapy for ASCC (NCT05060471).

The limitation of our study is obvious as it is a retrospective study with small sample in the single tertiary cancer center. Due to the long collection period of cases in this study, some cases data could only be collected from paper records. Partial data of clinical characteristics were missing. In addition, the specific strategies of CRT for patients with ASCC enrolled in our study were inconsistent.

In conclusion, CRT is the first choice for the treatment strategy of M0 stage ASCC. Induction chemotherapy may bring better OS and PFS benefits for bulk and locally advanced patients. The prognosis of M0 patients is heterogeneous due to the primary site of the tumor and T stage of the tumor, and more individualized treatment strategies need to be further explored. Patients with ASCC in China seem to have a better local control rate, which needs to be further verified by multicenter and larger sample size data.

## ETHICS STATEMENT

This study was approved by the Medical Ethics Committee of Sun Yat‐sen University Cancer Center (IRB B2020‐125‐01).

## CONFLICT OF INTEREST

The authors declare they have no conflict of interest.

## AUTHORS’ CONTRIBUTIONS

W.W.X. and Q.X.W. had the original idea for the study. W.W.X. had access to all data in the study and was responsible for the integrity of the data and the accuracy of the data analyses. Y.Y., W.H.X., and R.Z.L. participated in analyzing the data and writing of the manuscript. H.C. and Z.F.Z. prepared figures and tables. Y.H.G. revised the manuscript. All authors have read, edited, and approved the final version of the manuscript.

## Supporting information

Supplementary Material

## Data Availability

All data are available via the corresponding author.

## References

[1] Bray F , Ferlay J , Soerjomataram I , Siegel RL , Torre LA , Jemal A . Global cancer statistics 2018: GLOBOCAN estimates of incidence and mortality worldwide for 36 cancers in 185 countries. CA Cancer J Clin. 2018;68(6):394‐424. 30207593 10.3322/caac.21492

[2] Chen W , Zheng R , Baade PD , et al. Cancer statistics in China, 2015. CA Cancer J Clin. 2016;66(2):115‐132. 26808342 10.3322/caac.21338

[3] Hoots BE , Palefsky JM , Pimenta JM , Smith JS . Human papillomavirus type distribution in anal cancer and anal intraepithelial lesions. Int J Cancer. 2009;124(10):2375‐2383. 19189402 10.1002/ijc.24215

[4] Kobayashi T , Sigel K , Kalir T , MacLeod IJ , Liu Y , Gaisa M . Anal Cancer precursor lesions in HIV‐infected persons: tissue human papillomavirus type distribution and impact on treatment response. Dis Colon Rectum. 2019;62(5):579‐585. 30570548 10.1097/DCR.0000000000001307

[5] Lin C , Franceschi S , Clifford GM . Human papillomavirus types from infection to cancer in the anus, according to sex and HIV status: a systematic review and meta‐analysis. Lancet Infect Dis. 2018;18(2):198‐206. 29158102 10.1016/S1473-3099(17)30653-9 PMC5805865

[6] Du H , Luo H , Wang C , Qu X , Belinson JL , Wu R . The prevalence of HR‐HPV infection based on self‐sampling among women in China exhibited some unique epidemiologic features: HPV infection of Chinese based on self‐sampling. J Clin Epidemiol. 2021. [Online ahead of print]. 10.1016/j.jclinepi.2021.06.009 34161804

[7] Jones CM , Goh V , Sebag‐Montefiore D , Gilbert DC . Biomarkers in anal cancer: from biological understanding to stratified treatment. Br J Cancer. 2017;116(2):156‐162. 27923035 10.1038/bjc.2016.398 PMC5243987

[8] Nigro ND , Vaitkevicius VK Jr , Considine B . Combined therapy for cancer of the anal canal: a preliminary report. Dis Colon Rectum. 1974;17(3):354‐356. 10.1007/BF02586980 4830803

[9] NCCN Clinical Practice Guidelines in Oncology (NCCN Guidelines:Anal Carcinoma). 2020.

[10] Bartelink H , Roelofsen F , Eschwege F , et al. Concomitant radiotherapy and chemotherapy is superior to radiotherapy alone in the treatment of locally advanced anal cancer: results of a phase III randomized trial of the European Organization for Research and Treatment of Cancer Radiotherapy and Gastrointestinal Cooperative Groups. J Clin Oncol. 1997;15(5). 10.1200/JCO.1997.15.5.2040 9164216

[11] Northover J , Glynne‐Jones R , Sebag‐Montefiore D , et al. Chemoradiation for the treatment of epidermoid anal cancer: 13‐year follow‐up of the first randomised UKCCCR Anal Cancer Trial (ACT I). Br J Cancer. 2010;102(7):1123‐1128. 20354531 10.1038/sj.bjc.6605605 PMC2853094

[12] Rao S , Sclafani F , Eng C , et al. International rare cancers initiative multicenter randomized phase II trial of cisplatin and fluorouracil versus carboplatin and paclitaxel in advanced anal cancer: InterAAct. J Clin Oncol. 2020;38(22):2510‐2518. 32530769 10.1200/JCO.19.03266 PMC7406334

[13] Epidermoid anal cancer: results from the UKCCCR randomised trial of radiotherapy alone versus radiotherapy, 5‐fluorouracil, and mitomycin. UKCCCR Anal Cancer Trial Working Party. UK Co‐ordinating Committee on Cancer Research. Lancet. 1996;348(9034):1049‐1054. 8874455

[14] Carr R , Jin Z , Hubbard J . Research on anal squamous cell carcinoma: systemic therapy strategies for anal cancer. Cancers. 2021;13(9):2180. 34062753 10.3390/cancers13092180 PMC8125190

[15] Lu Y , Wang X , Li P , et al. Clinical characteristics and prognosis of anal squamous cell carcinoma: a retrospective audit of 144 patients from 11 cancer hospitals in southern China. BMC Cancer. 2020;20(1):679. 32693779 10.1186/s12885-020-07170-z PMC7372759

[16] Dindo D , Demartines N , Clavien PA . Classification of surgical complications: a new proposal with evaluation in a cohort of 6336 patients and results of a survey. Ann Surg. 2004;240(2):205‐213. 15273542 10.1097/01.sla.0000133083.54934.ae PMC1360123

[17] Škamperle M , Kocjan BJ , Maver PJ , Seme K , Poljak M . Human papillomavirus (HPV) prevalence and HPV type distribution in cervical, vulvar, and anal cancers in central and eastern Europe. Acta Dermatovenerol Alp Pannonica Adriat. 2013;22(1):1‐5. 23674179

[18] Liu W , Geng J , Fan Z , et al. Gene analysis of human papillomavirus 16/18 infection in anorectal and colorectal malignancies. Chin J Med Postgraduates. 2011;24(10):30‐34.

[19] Ajani JA , Winter KA , Gunderson LL , et al. Fluorouracil, mitomycin, and radiotherapy vs fluorouracil, cisplatin, and radiotherapy for carcinoma of the anal canal: a randomized controlled trial. JAMA. 2008;299(16):1914‐1921. 18430910 10.1001/jama.299.16.1914

[20] Brabin L . Interactions of the female hormonal environment, susceptibility to viral infections, and disease progression. AIDS Patient Care STDS. 2002;16:211‐221. 12055029 10.1089/10872910252972267

[21] Li Y , Lin J , Wu H , et al. Novel methotrexate prodrug‐targeted drug delivery system based on PEG‐lipid‐PLA hybrid nanoparticles for enhanced anticancer efficacy and reduced toxicity of mitomycin C. J Mater Chem B. 2014;2(38):6534‐6548. 32261815 10.1039/c4tb00499j

[22] Yoshimoto M , Saito M , Tada T , Takahashi K , Kasumi F . Unexpected increase in the bone marrow toxicity of mitomycin C (MMC). Br J Cancer. 2001;84(5):736‐737. 11237399 10.1054/bjoc.2000.1644 PMC2363787

[23] Okuno SH , Frytak S . Mitomycin lung toxicity. Acute and chronic phases. Am J Clin Oncol. 1997;20(3):282‐284. 9167754 10.1097/00000421-199706000-00015

[24] Flam M , John M , Pajak TF , et al. Role of mitomycin in combination with fluorouracil and radiotherapy, and of salvage chemoradiation in the definitive nonsurgical treatment of epidermoid carcinoma of the anal canal: results of a phase III randomized intergroup study. J Clin Oncol. 1996;14(9):2527‐2539. 8823332 10.1200/JCO.1996.14.9.2527

[25] Gunderson LL , Winter KA , Ajani JA , et al. Long‐term update of US GI intergroup RTOG 98–11 phase III trial for anal carcinoma: survival, relapse, and colostomy failure with concurrent chemoradiation involving fluorouracil/mitomycin versus fluorouracil/cisplatin. J Clin Oncol. 2012;30(35):4344‐4351. 23150707 10.1200/JCO.2012.43.8085 PMC3515768

[26] Li J , Xu H , Zou J , Wang X , Li Z , Shen Y . Cisplatin/capecitabine with intensity‐modulated radiation therapy in anal squamous cell carcinoma: a preliminary study. Scand J Gastroenterol. 2021;56(4):432‐436. 33556252 10.1080/00365521.2021.1879250

[27] Sun P , Li Y‐H , Wang W , Chen C‐Q , Wang L‐Y . Malignancies of the anal canal: a multi‐center retrospective analysis in South China population. J BUON. 2014;19(1):103‐108. 24659650

[28] Olivatto LO , Cabral V , Rosa A , et al. Mitomycin‐C‐ or cisplatin‐based chemoradiotherapy for anal canal carcinoma: long‐term results. Int J Radiat Oncol Biol Phys. 2011;79(2):490‐495. 20472349 10.1016/j.ijrobp.2009.11.057

[29] James RD , Glynne‐Jones R , Meadows HM , et al. Mitomycin or cisplatin chemoradiation with or without maintenance chemotherapy for treatment of squamous‐cell carcinoma of the anus (ACT II): a randomised, phase 3, open‐label, 2 × 2 factorial trial. Lancet Oncol. 2013;14(6):516‐524. 23578724 10.1016/S1470-2045(13)70086-X

[30] Chakravarthy AB , Catalano PJ , Martenson JA , et al. Long‐term follow‐up of a Phase II trial of high‐dose radiation with concurrent 5‐fluorouracil and cisplatin in patients with anal cancer (ECOG E4292). Int J Radiat Oncol Biol Phys. 2011;81(4):e607‐e613. 21514072 10.1016/j.ijrobp.2011.02.042 PMC3197794

[31] Ben‐Josef E , Moughan J , Ajani JA , et al. Impact of overall treatment time on survival and local control in patients with anal cancer: a pooled data analysis of Radiation Therapy Oncology Group trials 87–04 and 98–11. J Clin Oncol. 2010;28(34):5061‐5066. 20956625 10.1200/JCO.2010.29.1351 PMC3018356

[32] Nilsson PJ , Svensson C , Goldman S , Ljungqvist O , Glimelius B . Epidermoid anal cancer: a review of a population‐based series of 308 consecutive patients treated according to prospective protocols. Int J Radiat Oncol Biol Phys. 2005;61(1):92‐102. 15629599 10.1016/j.ijrobp.2004.03.034

[33] Moureau‐Zabotto L , Viret F , Giovaninni M , et al. Is neoadjuvant chemotherapy prior to radio‐chemotherapy beneficial in T4 anal carcinoma? J Surg Oncol. 2011;104(1):66‐71. 21240983 10.1002/jso.21866

[34] Mirghani H , Amen F , Blanchard P , et al. Treatment de‐escalation in HPV‐positive oropharyngeal carcinoma: ongoing trials, critical issues and perspectives. Int J Cancer. 2015;136(7):1494‐1503. 24622970 10.1002/ijc.28847

[35] Ott PA , Piha‐Paul SA , Munster P , et al. Safety and antitumor activity of the anti‐PD‐1 antibody pembrolizumab in patients with recurrent carcinoma of the anal canal. Ann Oncol. 2017;28(5):1036‐1041. 28453692 10.1093/annonc/mdx029 PMC5406758

[36] Morris VK , Salem ME , Nimeiri H , et al. Nivolumab for previously treated unresectable metastatic anal cancer (NCI9673): a multicentre, single‐arm, phase 2 study. Lancet Oncol. 2017;18(4):446‐453. 28223062 10.1016/S1470-2045(17)30104-3 PMC5809128

[37] Shahjehan F , Kamatham S , Ritter A , Kasi PM . Dramatic response to modified docetaxel, cisplatin, and fluorouracil chemotherapy after immunotherapy in a patient with refractory metastatic anal cancer. Clin Case Rep. 2019;7(9):1729‐1734. 31534737 10.1002/ccr3.2333 PMC6745381

