# Supplementary material for: Comprehensive treatment experience of anal squamous cell carcinoma from a tertiary cancer center in South China
Source: Cancer Med. 2021 Nov 24;11(1):117–27. doi: 10.1002/cam4.4433 (PMC8704146; doi:10.1002/cam4.4433)
Supplement: Supplementary file 1 — Supplementary Material [file CAM4-11-117-s001.docx]

**Supplementary Materials**

Supplementary Table 1. Correlation matrix analysis.

Note: **, strong correlation between two variables.

Supplementary Figure 1. Number of cases diagnosed at different time periods.


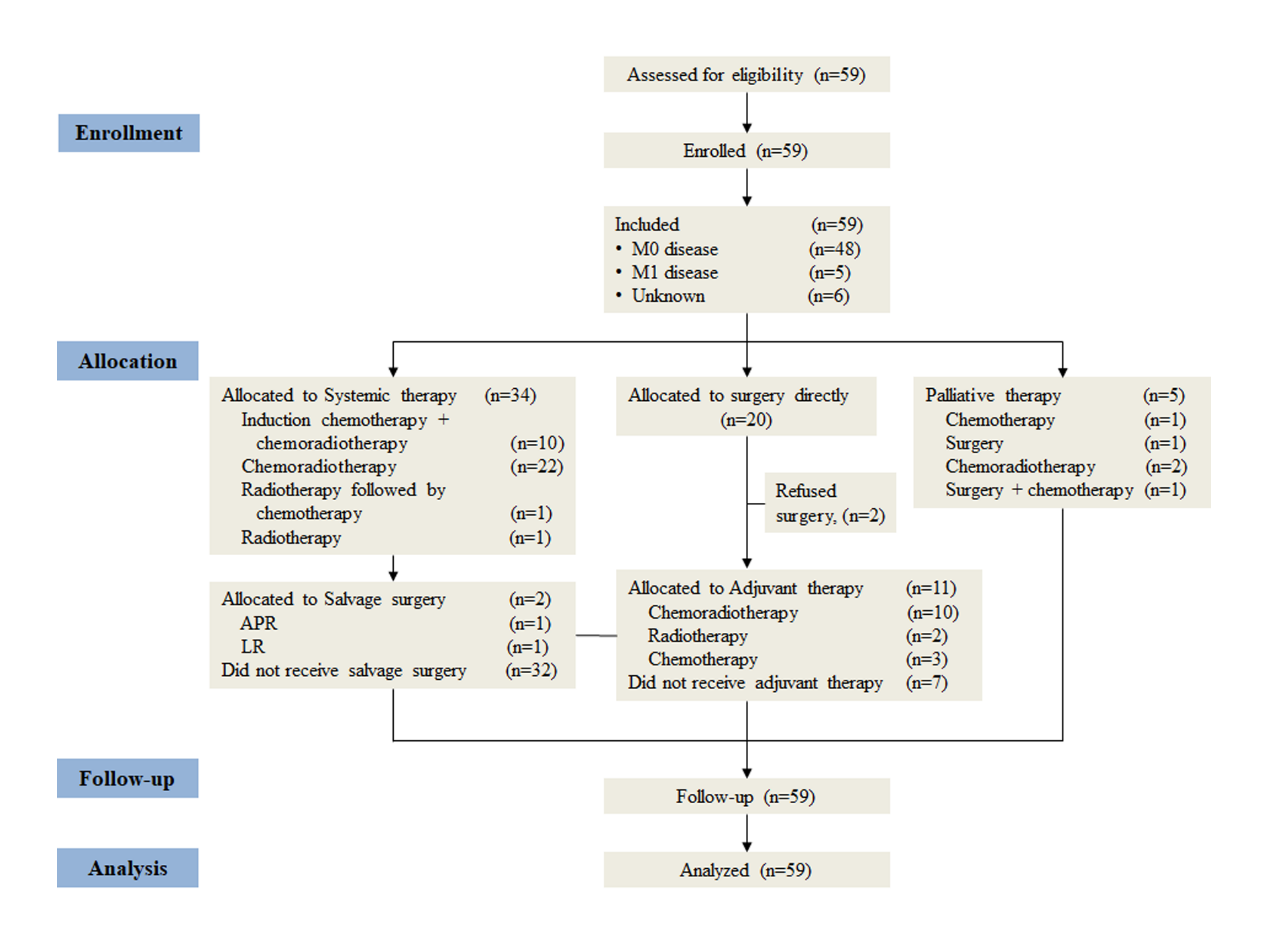


**Abbreviations:** APR, abdominoperineal resection; LR, local resection.

Supplementary Figure 2. Number of cases diagnosed at different time periods.

Supplementary Figure 3. Subgroup analysis of Survival. OS (A), PFS (B). P value was calculated.


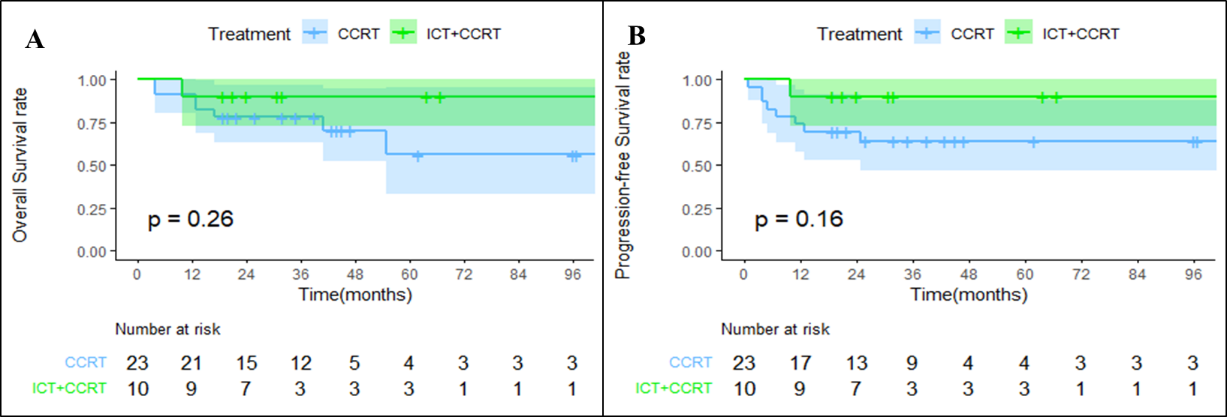


**Abbreviations:** ICT, induction chemotherapy; CCRT, concurrent chemoradiotherapy.
